# Statuses of food-derived glutathione in intestine, blood, and liver of rat

**DOI:** 10.1038/s41538-018-0011-y

**Published:** 2018-02-06

**Authors:** Hiroaki Yamada, Shinn Ono, Sayori Wada, Wataru Aoi, Eun Young Park, Yasushi Nakamura, Kenji Sato

**Affiliations:** 1 Division of Applied Biosciences, Graduate School of Agriculture, Kyoto University, Kitashirakawa Oiwake-cho, Kyoto, 606 8502 Japan; 2 College of Bioscience and Chemistry, Kanazawa Institute for Technology, Yatsukaho 3-1, Hakusann, 924 0838 Japan; 3 Division of Applied Life Sciences, Graduate School of Life and Environmental Sciences, Kyoto Prefectural University, Hangi-cho Shimogamo, Kyoto, 606 8522 Japan; 4 Present Address: Department of Food and Nutrition, Korea Christian University, 47, Kkachisan-ro 24-gil, Gangseo-gu, Seoul 07661 Korea

**Keywords:** Peptides, Peptides, Peptides, Peptides

## Abstract

Oral administration of glutathione has been demonstrated to reduce exercise-induced fatigue and improve liver function, although glutathione can be synthesized in the liver. However, little is known about the underlying mechanism of this effect. To address this, the status of food-derived glutathione in the intestine, blood, and liver was examined. Glutathione-1-^13^C or *N*-acetyl-cysteine-1-^13^C (NAC) was orally administered to rats (50 mg/kg). Food-derived glutathione contents within tissues were estimated by subtracting endogenous glutathione-1-^13^C from the total glutathione-1-^13^C. Food-derived glutathione was present in rat intestines and livers (approximately 60 and 300 μmol/kg, respectively, 120 min after ingestion) in electrochemically reduced form, while all food-derived glutathione in the blood plasma was conjugated with proteins and low-molecular-weight thiol compounds. However, no significant amounts of NAC-derived glutathione were detected in the blood plasma. These findings indicate that food-derived glutathione is directly absorbed in its electrochemically reduced form in the intestine, is then transported in the blood in bound forms, and is finally deposited into the liver in reduced form. Therefore, upon entering the bloodstream, food-derived glutathione binds to thiol compounds and releases hydrogen atom; subsequently, it does the reverse upon incorporation into the liver, which might impact the physiological redox condition. With respect to food-derived glutathione and cysteine-containing peptides, this study provides new insights on their modes of transportation and mechanisms of action.

## Dietary supplement: understand the benefits of glutathione

Though human body itself can synthesize glutathione, oral administration of the glutathione supplements was shown to be effective on whitening skin, reducing fatigue and improving liver function. To understand the mechanism of these benefits, Kenji Sato from Kyoto University and coworkers examined the circulation and conversion of food-derived glutathione. They found glutathione conjugated to thiol groups of protein and other low-molecular-weight compounds in blood plasma while ended up incorporating into the liver. They proposed this conversion between bound- and free-form glutathione released and received hydrogen atom and thiol compounds, which can activate the antioxidant enzymes and enhance the mitochondrial biogenesis, leading to these known beneficial effects. The present work may encourage further studies on the transportation and incorporation of food-derived glutathione and food proteins.

## Introduction

Glutathione (γ-L-glutamyl-L-cysteinyl-glycine) is widely distributed in known life forms, from single-cell organisms to higher vertebrates, and is the most prevalent intracellular thiol.^1,2^ Glutathione is also a cofactor or substrate of glutathione peroxidase and glutathione S-transferase. Glutathione peroxidase catalyzes the reduction of hydrogen peroxide and lipid peroxides into water and hydroxyl lipids, respectively, along with the oxidation of glutathione (reduced-form) into glutathione disulfide. Glutathione peroxidase plays a crucial role in maintaining proper physiological redox conditions and protecting proteins, lipids, and nucleic acids from oxidative stress. Glutathione is regenerated from glutathione disulfide by the enzyme glutathione reductase. Glutathione-S-transferase catalyzes the conjugation of glutathione and a wide variety of electrophilic compounds (both endogenous and exogenous): this is the first step in the mercapturic acid pathway, a crucial component of system detoxification.

Glutathione can be synthesized in the cells via a two-step ATP-dependent enzymatic process; the first step is conjugation of glutamic acid and cysteine, which is catalyzed by glutamate-cysteine ligase and generates γ-glutamyl-cysteine (γ-Glu-Cys) and the second step is catalyzed by glutathione synthase, which adds glycine to γ-Glu-Cys, forming glutathione.^1,2^ Glutathione is present in many foods and has been produced by fermentation to be used as supplement for whitening the skin, moderating fatigue, and improving liver functions in some countries. Oral administration of glutathione in the treatment group has been reported to reduce melanin indices on the skin^3^ and exercise-induced fatigue compared to that in the placebo controls.^4^ Recently, oral administration of glutathione has been demonstrated to significantly reduce plasma alanine aminotransferase and free fatty acid levels of patients with non-alcoholic fatty liver disease.^5^ These studies suggested that oral administration of glutathione was beneficial. Another study in humans demonstrated that dietary supplementation of glutathione resulted in an increase in protein-bound glutathione in the blood plasma,^6^ indicating that glutathione in foods and supplements can affect the blood glutathione level. However, glutathione doses used in these human trials were not that high (300–1000 mg/day). Small pieces (10–50 g) of meats, eggs, fish, and other animal foods generally contain sulfur-containing amino acids equivalent to cysteine in 300 mg glutathione. Therefore, ingestion of 300 mg of glutathione unlikely provides beneficial effects on liver function by only supplying glutathione or its precursor cysteine. However, glutathione can bind to thiol groups of protein and other low-molecular-weight compounds. Conversion between bound- and free-form glutathione releases and receives hydrogen atom and thiol compounds, respectively, which may induce physiological responses. However, the status of food-derived glutathione in hepatic portal blood—and upon its deposit into the target organ such as the liver—remain poorly understood.

The objectives of this study were to confirm the deposition of food-derived glutathione in the rat liver and to determine the status of food-derived glutathione in the intestines, blood, and liver using stable-isotope-labeled glutathione-1-^13^C and *N*-acetylcysteine-1-^13^C to better understand the mechanisms for beneficial effects of oral glutathione administration.

## Results and discussion

### Presence of food-derived glutathione in the body

Our previous study demonstrated that protein-bound glutathione in human plasma increases after ingestion of glutathione.^6^ However, whether the increased glutathione levels were directly derived from the ingested glutathione or synthesized from endogenous sources remains unclear.

**Fig. 1:**
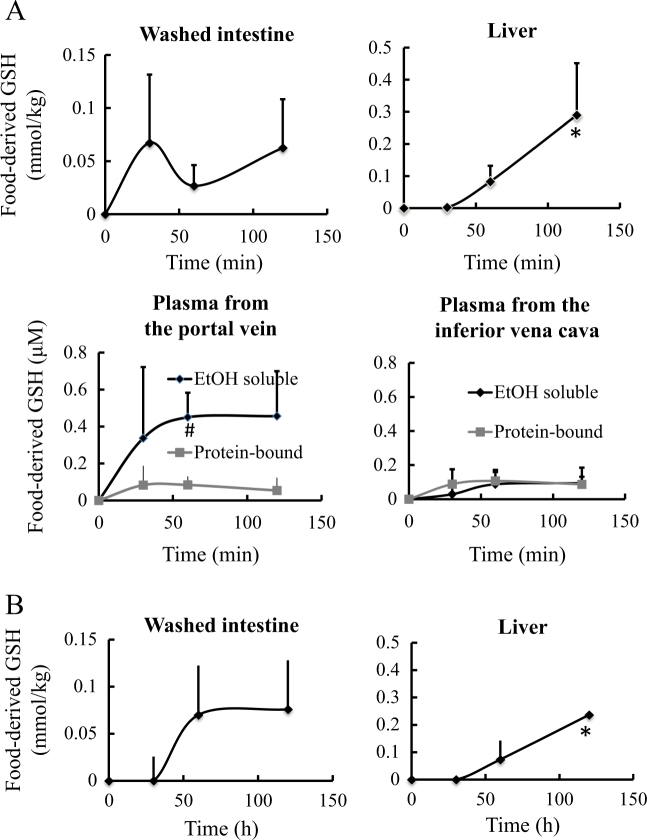
Total food-derived glutathione (GSH) contents in the washed intestine, liver, and low-molecular-weight (EtOH-soluble) and protein-bound fractions of blood plasma obtained from the portal and inferior vena cava. **a** After ingestion of stable isotope-labeled glutathione; **b** after ingestion of stable isotope-labeled *N*-acetylcysteine. Data are presented as mean ± standard deviation. * indicates significant difference compared with levels before ingestion (*P* < 0.05). # indicates significant difference compared with the counterpart from the inferior vena cava

**Fig. 2:**
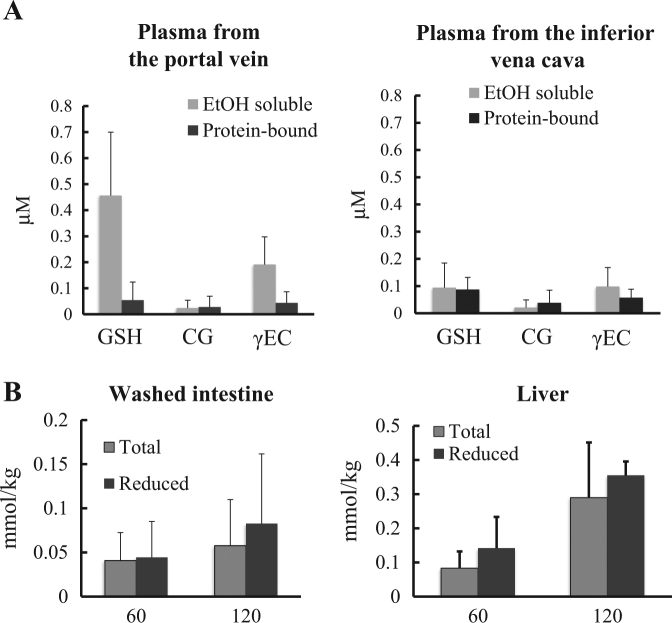
Contents of total food-derived glutathione (GSH) and its fragments, cysteinyl-glycine (CG), and gamma-glutamyl-glycine (γ-EC) in the blood plasma (**a**) and status of food-derived glutathione in washed intestine and liver (**b**). **a** Total glutathione and fragment peptides in low-molecular-weight (EtOH-soluble) and protein-bound fractions of the plasma 120 min after the ingestion. **b** Levels of total and reduced-form of glutathione in the washed intestine and liver 60 and 120 min after ingestion, respectively

To detect food-derived glutathione, all forms of food-derived glutathione were converted to a double conjugate with 6-aminoquinolyl-*N*-hydroxysuccinimidyl carbamate (AccQ) and 2-mercaptoethanol. As shown in Fig. 1, after ingestion of glutathione-1-^13^C, food-derived glutathione was detected in the intestine (approximately 60 μmol/kg at 120 min), liver (approximately 300 μmol/kg at 120 min), and blood plasma (both in low-molecular-weight and protein-bound fractions) of experimental rats. The difference between total glutathione as AccQ-2-mercaptoethanol double conjugate and reduced-form glutathione in the intestines and liver was insignificant, indicating that most of the food-derived glutathione in the intestine and liver were in the reduced form (Figs. 1 and 2). In the previous study, food-derived glutathione disulfide was, however, detected in liver and blood red cell of mice. However, blood in the liver used in the previous study was not purged. Then, the glutathione disulfides in the mice liver might be derived from the red cell.^7^ In the intestines and liver, only negligible amounts of food-derived cysteinyl-glycine (Cys-Gly) and gamma-glutamyl-cysteine (γ-Glu-Cys), (a glutathione-degradation product and a precursor of glutathione, respectively) were detected, while significant amounts of food-derived Cys-Gly and γ-Glu-Cys were detected in the plasma. After ingestion of *N*-acetylcysteine-1-^13^C (NAC), food-derived glutathione was also present in the intestines and liver, indicating that NAC can be used for glutathione synthesis in these organs (Fig. 1b), whereas NAC-derived glutathione and its fragment peptides were not detected in the plasma. Then, at least within 120 min, the endogenously synthesized glutathione in intestinal and liver cells was not excreted into the bloodstream. The presence of food-derived glutathione in the portal blood after ingestion of stable isotope-labeled glutathione indicates that it passes through the lining of the intestinal tract, which is consistent with our previous in vitro studies using Caco-2 cell line (a widely used model for testing the intestinal permeability of compounds) and small intestines mounted on an Ussing chamber.^7^

### Status of food-derived glutathione in the blood plasma

**Fig. 3:**
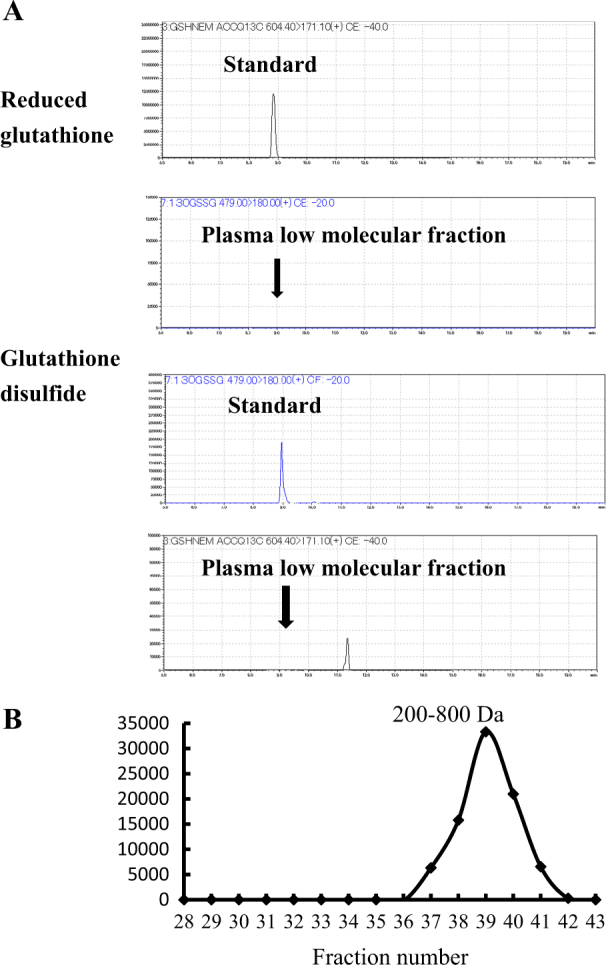
Status of glutathione in low-molecular-weight fraction of blood plasma obtained from the portal vein. **a** Detection of reduced-form of glutathione and glutathione disulfide using the LC-MS/MS in multi-reaction monitoring mode. The arrow indicates elution position of the standards. No glutathione and glutathione disulfide were detected. **b** Detection of glutathione in size exclusion chromatography fractions of low-molecular-weight fraction of blood plasma after reduction with 2-mercaptoethanol

Food-derived Cys-Gly and γ-Glu-Cys were detected in the blood plasma (Fig. 2a), indicating that at least a portion of food-derived glutathione is degraded by γ-glutamyltransferase or other peptidases (either circulating freely in the blood or on the surface of cell membranes) during blood circulation. As shown in Fig. 1a, 60 min after ingestion, food-derived glutathione levels in the low-molecular-weight fraction of blood from the hepatic portal vein (approximately 0.4 μM) were significantly higher (*P* < 0.05) than those in the corresponding fraction from the inferior vena cava (<0.1 μM). The food-derived glutathione levels within the protein-bound fraction between the plasma from the portal vein and from the inferior vena cava were insignificantly different, which indicates that glutathione in the low-molecular-weight fraction may be preferentially incorporated into the liver. As shown in Fig. 3, no reduced-form glutathione and glutathione disulfide were detected in the low-molecular-weight fraction of plasma from the portal vein. These data agree with the recent studies using high-performance liquid chromatography (HPLC) coupled with electrochemical detection.^8^ In contrast, the presence of glutathione and its disulfide in the plasma has been suggested based on the detection of thiol group using Ellman’s reagent and glutathione reductase.^7,9^ However, as shown in Supplementary Figure, a couple of compounds with free thiol group and disulfide bond are present in the low-molecular-weight fraction of rat plasma, which can react with Ellman’s reagent and provide apparent values for glutathione and its disulfide. Furthermore, it has been demonstrated that glutathione reductase can react other disulfides in addition to glutathione disulfide.^10^ Therefore, to determine the low amounts of glutathione and its derivatives in the blood plasma, direct chromatographic separation and a specific detection method such as mass spectrometry are necessary.

The compounds in the low-molecular-weight fraction of the plasma from portal vein, which was collected 60 min after the ingestion of glutathione, were fractionated by size exclusion chromatography (SEC). As shown in Fig. 3b, upon reduction with 2-mercaptoethanol, glutathione was released between SEC fractions 37 and 41. The mass spectrometry analysis revealed that peptides with approximately 200–800 Da were eluted in these fractions. Consequently, orally administered glutathione is bound to other low-molecular-weight thiol compounds.

### Incorporation of food-derived glutathione into the liver

The present study demonstrates the deposition of reduced-form food-derived glutathione in the liver (Fig. 2b). In the liver without blood, only negligible amounts of food-derived Cys-Gly, γ-Glu-Cys, and glutathione disulfide were detected. Detailed mechanism regarding the transportation of plasma bound-form glutathione into the hepatocytes remains unknown. High contents (approximately 6 mmol/kg) of endogenous reduced-form glutathione were present in the rat liver. If the plasma bound-form glutathione were to be converted to the reduced form before passing through the cell membrane, it would have to be transported into hepatocytes containing extremely higher concentrations of the same compound. This kind of transporter system is not yet known. The bound-form glutathione is likely transported into the hepatocytes and then converted to the reduced form. Alternatively, the bound-form glutathione may be degraded into amino acids on the cell membrane surface and the resulting amino acids may be incorporated into the hepatocytes and then used for intracellular glutathione synthesis. In both cases, bound-form glutathione must be converted to reduced-form glutathione or cysteine on or in the hepatocytes. To elucidate the transportation system of the plasma bound-form glutathione into the hepatocytes, further studies using hepatocytes and labeled glutathione and its derivatives are ongoing.

### Possible mechanism for health-promoting effects of glutathione in food

Oral administration of glutathione has been reported to increase the liver glutathione level in animals by inhibiting endogenous glutathione synthesis.^11,12^ The present study also demonstrates that orally administered glutathione can be physiologically deposited in rat liver. Then, oral administration of glutathione can recover the acute shortage of liver glutathione in poisoning. However, food-derived glutathione in live was approximately 5% of the total glutathione in the liver 120 min after administration of glutathione at 50 mg/kg. Furthermore, our preliminary experiment revealed that long-term (12 weeks) supplementation of glutathione (50 mg/kg body weight) in mice did not significantly increase liver glutathione levels compared to than in controls, indicating that oral administration of glutathione does not significantly affect the glutathione levels in the liver, when sufficient amounts of glutathione can be synthesized. Therefore, beneficial effects of orally administered glutathione on exercise-induced fatigue^4^ and liver function^5^ cannot be explained simply by supplying glutathione or its precursors. Furthermore, our previous animal experiment demonstrated that glutathione enhances mitochondrial biogenesis by increasing peroxisome proliferator-activated receptor gamma coactivator 1-alpha (PGC-1α),^4^ which is increased by exercise and enhances mitochondrial biogenesis. However, anti-oxidants such as vitamins C and E suppress exercise-induced increase of PGC-1α.^13,14^ Therefore, orally administered glutathione increased PGC-1α through a non-antioxidant property. The present study demonstrates that reduced-form food-derived glutathione is present in the intestines and liver, but in bound-form in the blood plasma. Therefore, food-derived glutathione binds to other thiol compounds and releases hydrogen atom on entering the blood and also releases the thiol compounds and receives hydrogen atom on or in the hepatocytes. Then, food-derived glutathione induces mild oxidative stress upon entering the cell, which could elevate PGC-1α. Similarly, the released thiol compounds from glutathione upon entering the cell might react with the thiol groups on other proteins such as Kelch-like ECH-associated protein 1, which may enhance translocation of nuclear factors (erythroid-derived 2)-like 2 (NRF2) into the nuclei and expression of antioxidant enzymes.^15^ Based on the present results, we hypothesize that food-derived glutathione has beneficial effects on chronic diseases by activating the host antioxidant enzymatic systems and mitochondrial biogenesis through the hydrogen atom–thiol compound exchange reaction on glutathione. This hypothesis must be corroborated by further in vitro and in vivo studies.

Some food proteins such as lactoferrin consist of relatively high levels of cysteinyl residues. After the ingestion of these proteins, some cysteine-containing peptides may be generated in the intestinal tract. However, there is no available data on the absorption, transportation, and incorporation of food-derived cysteine-containing peptides. The present study encourages us to address this issue.

## Methods

### Chemicals

Reduced form of L-glutathione was obtained from KOHJIN Life Sciences (Tokyo, Japan). Glutathione disulfide, γ-L-glutamyl-L-cysteine, L-cysteinyl-glycine, and L-cystine with ^13^C isotopes at C1 and C1′ (L-Cystine-1,1′-^13^C) were purchased from Sigma-Aldrich (St. Louis, MO, USA). L-Cysteine was obtained from Wako Pure Chemicals (Osaka, Japan), 6-aminoquinolyl-*N*-hydroxysuccinimidyl carbamate (AccQ) from Synchem (Altenburg, Germany), and the AccQ. Tag Ultra Derivatization Kit from Waters (Milford, MA, USA). Acetonitrile (HPLC-grade), triethylamine, formic acid, trichloroacetic acid (TCA), 2-mercaptoethanol, and *N*-ethylmaleimide (NEM) were obtained from Nacalai Tesque (Kyoto, Japan). Amino acid derivatives and reagents for peptide synthesis were purchased from Watanabe Chemical (Hiroshima, Japan).

### Preparation of working reagents

KH_2_PO_4_ (0.2 M) and Na_2_PO_4_ (0.2 M) were mixed at a ratio of 40.5:9.5, (v/v), and the pH, if necessary, was adjusted to 7.4 by adding KH_2_PO_4_ or Na_2_HPO_4_ solutions drop-wise. The prepared phosphate buffer was mixed with 0.154 M NaCl solution at a ratio of 1:9 (v/v) and used as phosphate-buffered saline (PBS). Triethylamine, methanol, and water were mixed at a ratio of 2:7:1 (v/v) that was used as an alkaline solution.

### Synthesis of stable isotope-labeled glutathione

Stable isotope-labeled glutathione was synthesized by a liquid-phase method from cystine-1,1′-^13^C using the disulfide bond as a thiol-protecting group. Cystine-1,1′-^13^C was converted into the corresponding *N*,*N*′-*tert*-butyloxycarbonyl (Boc) protected form using di-*tert*-butyl dicarbonate. The Boc-protected cystine-1,1′-^13^C was reacted with two equivalents of glycine *tert*-butyl ester (OtBu) hydrochloride using 1-ethyl-3-(3-dimethylaminopropyl)carbodiimide monohydrochloride and 1-hydroxybenzotriazole to produce the N and C-protected dipeptidyl (Boc-Cys-Gly-OtBu) disulfide derivative. Cleavage of Boc groups with 4 M HCl/dioxane followed by a coupling reaction with the γ-carboxyl group of the *N*-hydroxysuccinimide active ester of Boc-Glu-α-OtBu resulted in the corresponding glutathione disulfide-1,1′-^13^C with fully protected N and C-terminals; glutathione disulfide-1,1′-^13^C was prepared by deprotection with TFA. Glutathione-1-^13^C was then released by reduction with dithiothreitol (DTT) in an aqueous solution. Finally, the obtained crude product was purified by preparative reversed-phase HPLC using a Cosmosil 5C18-AR-II (20 mm i.d. × 250 mm; Nacalai Tesque). The corresponding fraction was lyophilized, giving 169 mg of the desired product in 41% yield. The final product was characterized by analytical reversed-phase HPLC using a TSKgel ODS-100S (3.0 mm i.d. × 150 mm; Tosoh, Tokyo Japan) and electrospray ionization time-of-flight mass spectrometry with a Hitachi Nano Frontier liquid chromatography–mass spectrometry (LC-MS) system (Tokyo, Japan).

### Preparation of stable isotope labeled *N*-acetylcysteine (NAC)

NAC-1-^13^C was synthesized from cystine-1,1′-^13^C. The amino group of cystine-1,1′-^13^C was reacted with acetic anhydride to produce its acetylated derivative. NAC-1-^13^C was released by reduction with DTT and purified by the preparative reversed-phase HPLC as described above. The final product (698 mg) was obtained in 54% yield and characterized by the analytic reversed-phase HPLC and ^1^H NMR with a JEOL JNX-ECX500 spectrometer (Tokyo, Japan). Each analytical result was consistent with the proposed structure.

### Animal experiments

All animals were treated and cared in accordance with the National Institutes of Health’s guidelines for the use of experimental animals. All experimental procedures were approved by the Animal Care Committee of Kyoto Prefectural University (No. KPU260400-260421). Seven-week-old Wister rats (body weight 200–220 g) were housed in a 12-h light-dark cycle and allowed free access to standard rat chow and water. Rats were acclimatized for a week, divided into four groups, and were given 1 mL of glutathione-1-^13^C (50 mg/kg body weight) or NAC-1-^13^C (50 mg/kg body weight) solutions using an oral gavage. Before and 30, 60, and 120 min after the administration, the rats were sacrificed by puncturing the inferior vena cava under a pentobarbital sodium (40–50 mg/kg) anesthesia. The blood was collected from the portal vein and inferior vena cava using a heparinized syringe. The plasma was prepared by centrifugation at 800×*g* for 10 min at 4 °C. The liver and small intestines were taken from the rats. The intestines were flushed with about 40 mL of cold PBS to remove its contents. The blood in the liver was purged by infusing cold PBS into the portal vein. Samples were stored at −80 °C until use.

### Sample preparation

The plasma (100 μL) was mixed with three fold volumes of ethanol. The supernatant and precipitate obtained were harvested by centrifugation at 14,200×*g* at 4 °C for 10 min. The supernatant was referred to as the low-molecular-weight fraction of the plasma. Aliquots of the low-molecular-weight fraction (300 μL each) were dried under vacuum, and processed using two methods. Some dried aliquots were treated with 50 μL of 5% TCA-2% 2-mercaptoethanol solution for 30 min and used in determining the total glutathione and related peptides. Free and bound-form glutathione were bound to 2-mercaptoethnol.^6^ Other dried aliquots of the fraction (300 μL) were treated with 50 μL of 20 mM NEM in PBS for 10 min. Only free glutathione bound to NEM, as NEM has no reducing activity. The reaction was terminated by adding 50 μL of 5% TCA solution. The NEM-treated fraction was used in determination of the reduced-form of glutathione and glutathione disulfide.

The ethanol precipitate of the plasma was further washed with 200 μL of 75% (v/v) ethanol twice. The residue was collected by centrifugation at 14,200×*g* at 4 °C. Protein-bound glutathione and related peptides were liberated from the residue by extracting with 100 μL of 5% TCA-2% 2-mercaptoethanol with occasional agitation for 30 min. The supernatant obtained after centrifugation was used to determine the protein-bound form of glutathione and related peptides.^6^

Portions of the thoroughly washed intestines and livers were homogenized in the same weight of PBS using a BioMasher (Nippi, Tokyo, Japan). The obtained homogenates were then mixed with the same volumes of 10% (v/v) TCA-4% (v/v) 2-mercaptoethanol and allowed to stand for 30 min at 25°C. The supernatant was collected and used to determine the total amounts of glutathione and related peptides. Another set of intestines and livers were homogenized in the same volume of PBS containing 20 mM NEM and then allowed to stand for 10 min at 25 °C. The homogenate were further diluted with another three fold volumes of PBS without NEM, and mixed with the same volume of 10% (v/v) TCA. The supernatants and precipitates were harvested by centrifugation at 14,200×*g* for 10 min at 4 °C. The obtained supernatants were used to determine the reduced-form of glutathione and glutathione disulfide. The precipitate was washed with the same weight of 10% TCA three times, and the protein-bound form of glutathione was extracted with the same weight of 10% TCA-4% 2-mercaptoethanol as described above.

### Determination of glutathione and related peptides

Pretreatment using a 5% TCA–2% 2-mercaptoethnol solution resulted in conjugation of all forms of glutathione and related peptides with 2-mercaptoethnol via a disulfide bond.^6^ The 5% TCA–2% 2-mercaptoethanol extracts (50 μL) were dried under vacuum to remove excess amounts of 2-mercaptoethanol; thereafter, 20 μL of an alkaline solution was added to neutralize the extracts and they were again dried under vacuum. The residues were mixed with 20 μL of 20 mM HCl, 60 μL of the sodium borate buffer provided with the AccQ Tag Ultra Derivatization Kit, and 20 μL of 0.3% (w/v) AccQ acetonitrile solution and heated at 50 °C for 10 min to obtain their AccQ derivatives. Then, the reaction mixtures were diluted with adequate amounts of 5 mM sodium phosphate buffer (pH 7.4) containing 10% (v/v) acetonitrile and filtered using a 0.45-μm filter (Cosmonice filter W, Nacalai Tesque). The filtrates were subjected to LC-MS/MS using a 3200 QTRAP system (AB SCIEX, Framingham, MA) as previously described.^6^

Without 2-mercaptoethanol, the sulfhydryl group of glutathione was conjugated with NEM in PBS. The NEM-glutathione derivative was directly subjected to LC-MS/MS to determine the reduced-form of glutathione. With NEM, glutathione disulfide was conjugated with two molecules of AccQs, and the resultant AccQ derivative of glutathione disulfide was subjected to the LC-MS/MS as described above. Reduced-form glutathione (2 nmol) was added to the ethanol-soluble fraction of plasma from portal blood. The recovery of the reduced-form glutathione was 82.5 ± 5.6%. Thus, the reduced form glutathione is stable under this condition and also matrix effect on ionization can be neglected.

Endogenous glutathiones harboring either all ^12^C or one ^13^C in samples, which were collected before the ingestion of glutathione-1-^13^C, were determined. The ratio between endogenous glutathione containing one ^13^C and that containing ^12^C, [*R*(^13^C/^12^C)], was obtained. The content of food-derived glutathione was estimated by using the following equation:

Food-derived glutathione content = (content of glutathione with one ^13^C in sample collected after ingestion) – *R*(^13^C/^12^C) × (content of glutathione with all ^12^C in sample collected after ingestion).

### Statistics

Differences among the time points were evaluated using one-way analysis of variance (ANOVA) followed by Turkey’s test using GraphPad Prism 6 (GraphPad Software, San Diego, CA, USA). Differences between two groups were evaluated using *t*-test.

### Data availability

All relevant data are available from the corresponding author on reasonable request.

## Electronic supplementary material

Supplementary Figure

## Electronic supplementary material

**Supplementary information** accompanies the paper on the *npj Science of Food* website (10.1038/s41538-018-0011-y).
