# Supplementary material for: Statuses of food-derived glutathione in intestine, blood, and liver of rat
Source: NPJ Sci Food. 2018 Feb 6;2:3. doi: 10.1038/s41538-018-0011-y (PMC6550177; doi:10.1038/s41538-018-0011-y)
Supplement: Supplementary file 1 — Supplementary Figure [file 41538_2018_11_MOESM1_ESM.docx]

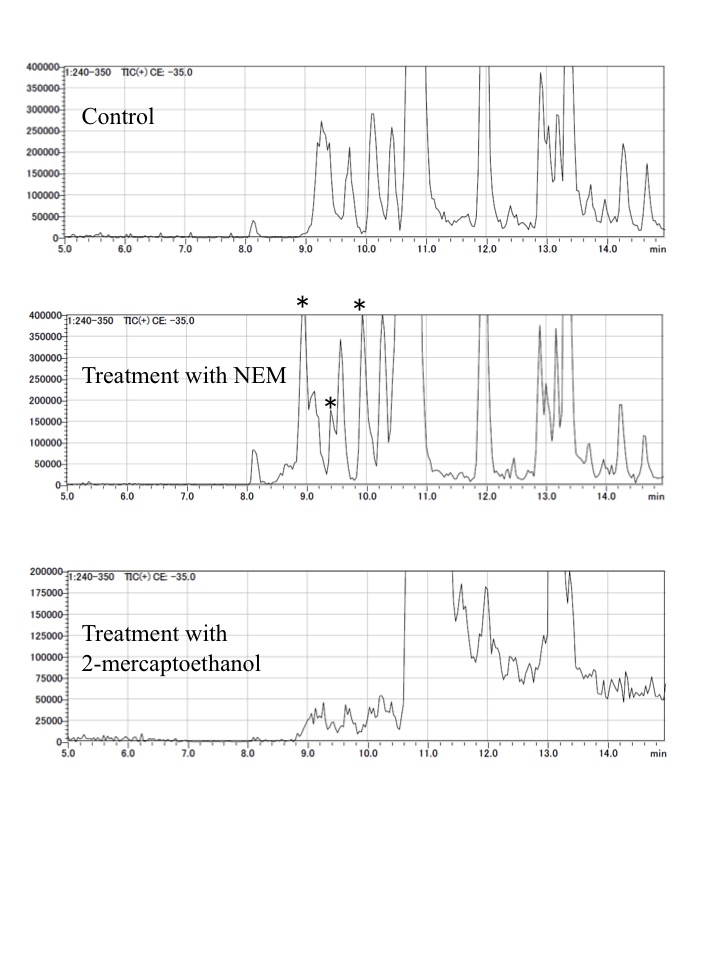


Supplementary Figure 1. LC-MS/MS chromatograms of low molecular weight fraction of plasma obtained from portal blood 60 min after ingestion of glutathione.

Two hundred micro litters of low molecular weight fraction of plasma was injected to size exclusion chromatography using Superdex Peptide 10/300 GL (GE Health Care, Little Chalfont, England) equilibrated with 0.1% (v/v) formic acid containing 10% (v/v) acetonitrile at 0.5 mL/min. Fractions were collected every minutes. Aliquots of SEC fractions were dried under vacuum and treated with *N*-ethylmaleimide (NEM) or 2% 2-mercaptoethamol followed by reaction with AccQ. The derivatives were subject to LC-MS/MS with same elution condition as described in Material and Method section using an LCMS-8040 (Shimadzu, Kyoto, Japan). The AccQ derivatives were specifically detected by precursor scan targeting AccQ-derived fragment ion (*m/z*=171.1). The peaks marked with asterisks appeared by treatment with NEM, which have free thiol group. By treatment with 2-mercaptoethanol, elution pattern changed extensively, indicating presence of disulfide bond and thiol group.
